# Novel protein isoforms of carcinoembryonic antigen are secreted from pancreatic, gastric and colorectal cancer cells

**DOI:** 10.1186/1756-0500-6-381

**Published:** 2013-09-26

**Authors:** Keiichi Hatakeyama, Kanako Wakabayashi-Nakao, Keiichi Ohshima, Naoki Sakura, Ken Yamaguchi, Tohru Mochizuki

**Affiliations:** 1 Medical Genetics Division, Shizuoka Cancer Center Research Institute, 1007 Shimonagakubo, Nagaizumi-cho, Sunto-gun, Shizuoka 411-8777, Japan; 2 Shizuoka Cancer Center Hospital and Research Institute, 1007 Shimonagakubo, Nagaizumi-cho, Sunto-gun, Shizuoka 411-8777, Japan

**Keywords:** CEA, Alternative splicing, Tumor marker, Splice variant, Protein isoform

## Abstract

**Background:**

Carcinoembryonic antigen-related cell adhesion molecule 5 (CEACAM5) is an oncofetal cell surface glycoprotein. Because of its high expression in cancer cells and secretion into serum, CEA has been widely used as a serum tumor marker. Although other members of CEACAM family were investigated for splice variants/variants-derived protein isoforms, few studies about the variants of *CEACAM5* have been reported. In this study, we demonstrated the existence of novel *CEACAM5* splice variants and splice variant-derived protein isoforms in gastrointestinal cancer cell lines.

**Results:**

We identified two novel *CEACAM5* splice variants in gastrointestinal (pancreatic, gastric, and colorectal) cancer cell lines. One of the variants possessed an alternative minor splice site that allowed generation of GC-AG intron. Furthermore, CEA protein isoforms derived from the novel splice variants were expressed in cancer cell lines and those protein isoforms were secreted into the culture medium. Although CEA protein isoforms always co-existed with the full-length protein, the secretion patterns of these isoforms did not correlate with the expression patterns.

**Conclusions:**

This is the first study to identify the expression of CEA isoforms derived from the novel splice variants processed on the unique splice site. In addition, we also revealed the secretion of those isoforms from gastrointestinal cancer cell lines. Our findings suggested that discrimination between the full-length and identified protein isoforms may improve the clinical utility of CEA as a tumor marker.

## Background

Carcinoembryonic antigen-related cell adhesion molecule 5 (CEACAM5; synonyms, CEA, CD66e) is a glycosylated oncofetal antigen and was first found in gastrointestinal cancer tissues [1,2]. The same research group that initially identified CEA subsequently indicated that CEA levels could be measured in serum from patients with colorectal and other carcinomas [3], which introduced the possibility of clinical application for diagnosis, monitoring and prognosis. CEA is now generally accepted as a valuable tumor marker for monitoring of several cancers following surgery.

CEA, which belongs to the CEACAM family of the immunoglobulin (Ig) superfamily [4,5], has been suggested to mediate cell adhesion on tumors [6-8], and the members of this family, i.e., CEACAM1, CEACAM3, CEACAM4, CEACAM6, CEACAM7, and CEACAM8, are also deregulated in various tumors. In this subset, splice variants and protein isoforms were previously identified, and among them, CEACAM1 was found to occur in various alternatively spliced forms [9]. Recently, Tamura et al. revealed that expression of CEACAM1 protein isoforms (CEACAM1-4L and -4S) was associated with cell adhesion in colorectal cancer cells [10]. Despite these findings, relatively few screening approaches for splice variants of CEA have been reported.

In normal colorectal tissues, CEA is actually expressed at the cell surface and released extracellularly. The expression of CEA is probably associated with anoikis as a surveillance mechanism to preserving the normal physiological architecture [11]. The secretion of this molecule that can bind to *E. coli* and *Salmonella* expressing type 1 fimbriae [12,13] is also considered to play an essential role in the host defense mechanism preventing the binding of pathogenic bacteria [14]. However, in cancerous cells, the role of the abundant release of CEA remains unclear.

To determine the expression and secretion patterns of CEA in gastrointestinal tumors, we demonstrated the existence of novel CEA splice variants and splice variant-derived protein isoforms in gastrointestinal cancer cell lines. Furthermore, these isoforms were found to be secreted to the culture medium. These findings suggested that the cancer-released CEA in the blood may include our identified protein isoforms.

## Methods

### Cell cultures and RNA sample preparations

Human gastrointestinal cancer cell lines QGP1, MKN45 and KATO III were purchased from the Japanese Collection of Research Bioresources, HPAC, HPAF II, SNU1 and LoVo from the American Type Culture Collection, and HCA2 and HCA46 from Dainippon Sumitomo Pharma (Osaka, Japan). These cell lines were cultured and maintained in RPMI 1640 medium (Sigma-Aldrich, St. Louis, MO) supplemented with 10% FBS (Invitrogen, Carlsbad, CA), glutamine (0.3 mg/ml), penicillin (100 unit/ml) and streptomycin (0.1 mg/ml) in a humidified 5% CO_2_ incubator.

After total RNA had been extracted from each pellet using the RNeasy Plus Mini Kit (Qiagen, Hilden, Germany), RNA concentration was determined using a NanoDrop spectrophotometer (Thermo Fisher Scientific, Waltham, MA) and total RNA quality was then confirmed using the Agilent 2100 Bioanalyzer (Agilent Technologies, Santa Clara, CA). The purified total RNA from cancer cell lines and total RNA derived from normal tissues (Clontech, Mountain View, CA) were then reverse-transcribed using ThermoScript Reverse Transcriptase (Invitrogen) and oligo(dT)_20_ primers in accordance with the manufacturer’s instructions.

### Reverse-transcriptase PCR

Screening of cDNA for different splice variants of *CEACAM5* was performed using the intron-spanning exonic primers listed in Additional file 1: Table S1. PCR amplicons were designed to detect the splice variants and the transcripts registered in GenBank (NM_004363). The synthesized cDNAs were amplified using LA Taq polymerase (Takara Bio, Shiga, Japan) by 35 PCR cycles of 95°C for 30 s, 51°C for 30 s, and 68°C for 60 s.

### Rapid amplification of cDNA ends and DNA sequencing analysis

To determine the sequence of novel *CEACAM5* transcripts, rapid amplification of cDNA ends (RACE) was conducted using the GeneRacer Kit (Invitrogen) according to the manufacturer’s instructions. The primer sequences are shown in Additional file 1: Table S1. For DNA sequencing analysis, the PCR products were analyzed on 1–2% agarose gels by electrophoresis following by gel staining with SYBR Safe (Invitrogen). The bands visualized under ultraviolet light were isolated and purified using the QIAquick Gel Extraction Kit (Qiagen). The purified samples were then cloned into a pCR 2.1-TOPO vector (Invitrogen) using the TOPO TA Cloning Kit for Sequencing (Invitrogen). Positive transformants were sequenced using the Big Dye Terminator v3.1 Cycle Sequencing Kit (Applied Biosystems, Foster City, CA) and an ABI 3130xl Genetic Analyzer (Applied Biosystems). The protein coding sequences obtained by the DNA sequencing have been deposited in DNA Data Bank of Japan (DDBJ), which are accessible through the DDBJ accession number AB852566 and AB852567.

### Quantitative real-time PCR

Quantitative real-time PCR (qRT-PCR) was performed using the SYBR Green dye technique and the ABI PRISM 7900HT Fast Real Time PCR System (Applied Biosystems, Foster City, CA). The target fragment was amplified using specific primers (Additional file 1: Table S1) according to the following protocol: preheating at 95°C for 20 s; 40 cycles at 95°C for 1 s and 60°C for 30 s; and dissociation at 95°C for 15 s, 60°C for 15 s, and 95°C for 15 s. The threshold cycle (Ct) values were converted into absolute copy numbers using a standard curve with purified PCR amplicons that were generated from plasmids containing the sequence of the target transcripts. Nonspecific amplification in qRT-PCR was evaluated using dissociation curves and gel electrophoresis that showed the amplicon size.

### Preparation of cell lysate and serum-free conditioned medium

Cells were placed (4 × 10^6^ cells) on 100-mm dishes and incubated until 70%–80% confluence. After incubation, the culture medium was removed and the cells were then harvested with ice-cold PBS. After centrifugation and removal of PBS, cell pellets were treated with lysis buffer containing 7.5 M urea, 2.5 M thiourea, 12.5% glycerol, 50 mM Tris–HCl (pH 7.4), 2.5% *N*-octylglucoside, 6.25 mM Tris-carboxyethyl phosphine hydrocholine (TCEP), and 1.25 mM protease inhibitor (Sigma-Aldrich, St. Louis, MO), and rotated at 4°C for 1 h. Following the rotation, the samples were centrifuged at 14,000 *× g* for 30 min at 4°C, and the supernatants were then collected. Protein concentrations were determined by the Bradford assay (BioRad Laboratories, Hercules, CA).

Serum-free conditioned medium was prepared as previously studied [15]. Briefly, cells were placed (8 × 10^6^ cells) on 100-mm dishes, and incubated at 37°C overnight. After removal of the conditioned medium, the cells were washed with PBS (pH 7.4) and then pre-incubated with 8 ml of serum-free medium at 37°C for 1 h. The conditioned medium was then removed and replaced with 3.5 ml of serum-free medium at 37°C for 24 h. The serum-free conditioned medium was collected and centrifuged to remove cell debris. The collected medium was then concentrated using Amicon Ultra Centrifugal Filters (4 mL, 10 K device, Millipore, Bedford, MA) at 4°C and 2,330 *× g*, and stored at −80°C until further analysis.

### Enzymatic deglycosylation

Cell lysates were purified using a 2-D Clean-Up Kit (GE Healthcare UK Ltd., Buckinghamshire, England) to precipitate proteins according to the manufacturer’s instructions. The resulting dry pellets were resuspended in 1× Glycoprotein Denaturing Buffer and incubated at 95°C for 10 min. The denatured samples were incubated with a reaction buffer containing 1× G7 Reaction Buffer, 1% NP-40, protease inhibitor, and PNGase F (New England Biolabs, San Leandro, CA) at 37°C for 1 h. The concentrated conditioned medium samples were also denatured and treated with PNGase F as described above. The deglycosylated samples were treated with SDS-PAGE sample buffer solution containing 200 mM DTT.

### Immunoblotting

CEA in the cell lysate or serum-free conditioned medium was detected by immunoblotting with an anti-CEA mouse monoclonal antibody (1:1,000 or 1:3,000 dilution; Clone C6G9, Sigma-Aldrich; this antibody was raised against the CEA isolated from a human colon adenocarcinoma cell line) as the primary antibody, and anti-mouse IgG-horseradish peroxidase (HRP) conjugate (1:3,000 or 1:10,000 dilution; Jackson ImmunoResearch, West Grove, PA) as the secondary antibody. HRP-dependent luminescence was developed using ECL Prime Western Blotting Detection Reagent (GE Healthcare) and detected using a LAS-3000 device (Fuji Film, Tokyo, Japan). To detect actin-ß, which was used as an internal loading control, immunoblot detection was conducted in the same manner described above, except for the use of a mouse monoclonal anti-actin-ß antibody (1:5,000 dilution; Sigma-Aldrich) as the primary antibody.

### Liquid chromatography-tandem mass spectrometry (LC-MS/MS) analysis

To identify the CEA isoforms, proteins were separated by SDS-PAGE and visualized by silver staining (SilverQuest^TM^ Silver Staining Kit, Invitrogen). Protein bands smearing around 90 kDa, 60 kDa, and 45 kDa were excised manually. Each gel slice was destained with Destainer solution in the staining kit and dehydrated with 100% acetonitrile. After drying under reduced pressure using the SpeedVac (Thermo Fisher Scientific), gel pieces were reduced with 50 μl of 10 mM DTT at 56°C for 45 min and alkylated with 50 μl of 55 mM iodoacetamide for 30 min at room temperature in the dark. The resulting samples were washed and dried using the SpeedVac, and then digested overnight with 8.3 ng/μl trypsin (Promega, Fitchburg, WI) at 37°C. The tryptic digests were purified with ZipTip C18 (Millipore), concentrated using the SpeedVac, and reconstituted in 0.1% formic acid.

LC-MS/MS analysis was conducted on a nanoflow LC-electrospray ionization linear ion trap-time of flight (LC-ESI LIT-TOF) mass spectrometer (NanoFrontier L; Hitachi High-Technologies, Tokyo, Japan). The methods used for each instrument and the strategy for data analysis are were previously described in detail [16].

Peptide sequences were identified against the SwissProt database (version: 2012_03) with the following parameters: enzyme, trypsin; maximum number of missed cleavages, 1; peptide tolerance, 0.4 Da; MS/MS tolerance, 0.4 Da; fixed modification, carbamidomethylation of cysteine; variable modification, oxidation of methionine and conversion of asparagine to aspartic acid; peptide charge, 1+, 2+, and 3+. The validity of a formerly *N*-glycosylated peptide was confirmed by the presence of a consensus N-X-S/T (X ≠ P) sequence and deamidation of the asparagine residues.

## Results

### Identification of novel *CEACAM5* exons

**Figure 1:**
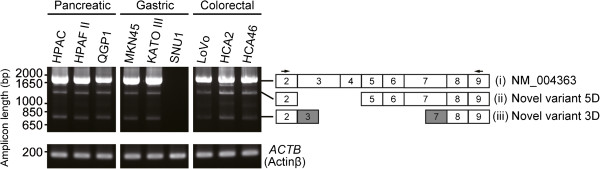
**Identification of *CEACAM5 transcripts in pancreatic, gastric and colorectal cancer cells.*** Electrophoresis images of RT-PCR are shown in the left panel. RT-PCR of *ACTB* was used as a positive control. Schematic diagram of exon structures corresponding to the detected amplicons are represented in the right panel. Novel variant 5D (ii) skipped exons 3 and 4 of full-length transcript NM_004363 (i). Novel variant 3D (iii) contained novel alternative splice forms (gray boxes), and skipped exons 4–6 in (i). Primer positions for RT-PCR are indicated by black arrows.

Expression of CEA is frequently high in gastrointestinal cancer [9]. RT-PCR screening of gastrointestinal (pancreatic, gastric, and colorectal) cancer cell lines was conducted using newly designed RT-PCR primers for several exons on the target gene and a primer pair spanning the region from exon 2 to exon 9 on the full-length *CEACAM5* registered in GenBank (NM_004363; Figure 1). RT-PCR screening revealed a positive signal (i) in eight cell lines, while two other bands (ii) and (iii) were also observed in those cell lines. To determine the exon structures of these amplicons, DNA sequencing was performed using RACE (data not shown). We identified two novel splice variants in gastrointestinal cancer cells, which were designated as variant 5D and 3D (Figure 1). The sequence of positive signal (i) was identical to that of NM_004363. The exon structure of variant 5D skipped exons 3–4, and variant 3D was truncated at the 3′ end of exon 3 and the 5′ end of exon 7 relative to the full-length transcript. Although the RT-PCR screening slightly detected bands other than (i)–(iii), novel alternatively spliced transcripts except variant 5D and 3D were not identified from these bands.

### Characterization of splice variant 3D

**Figure 2:**
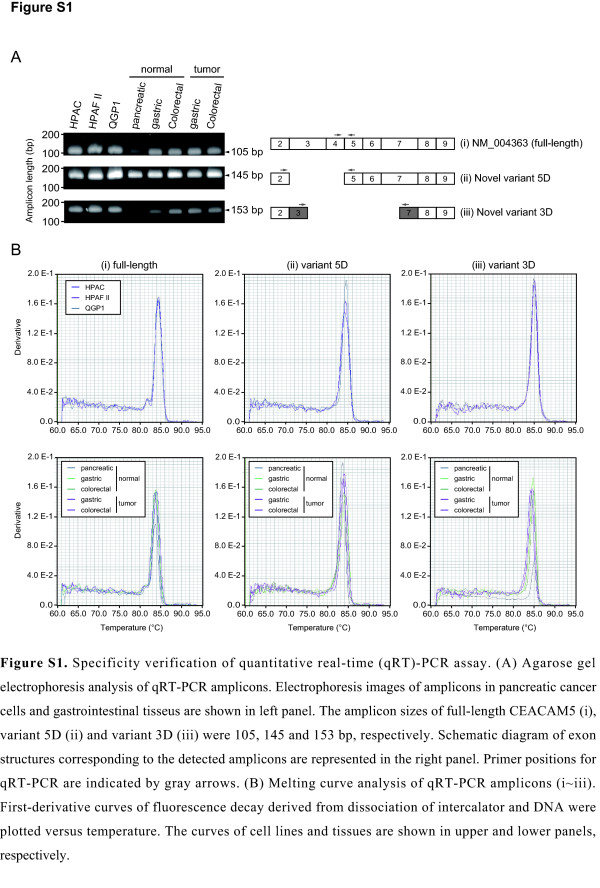
**Sequence analysis of novel variant 3D. A**, Part of the transcript sequence was shared between exons 3 and 7 in novel variant 3D. The shared bases are shown in bold characters. Regions that matched with exon 3 and 7 are indicated by solid and dashed line, respectively. Uppercase bases (AGGC) indicate potential splice site that allow the generation of GC-AG introns. **B**, Prediction of splice site between novel exons 3 and 7. Exons and introns are indicated in gray and white boxes, respectively. Variant 3D and GC-AG introns were generated by alternative splicing of the potential splice site.

Our RT-PCR and sequencing analysis revealed the presence of mRNA with alternative splicing patterns for *CEACAM5*. In variant 3D, we found a shared sequence between exons 3 and 7 (Figure 2A). A major splice site that can generate GT-AG introns was not included, but this shared region possessed an alternative minor splice site that allowed the generation of GC-AG introns [17]. Based on this finding, we predicted that the 5′- and 3′-terminal sequences of the intron would be between exons 3 and 7 in variant 3D (Figure 2B). Variant 3D was thus probably spliced at the potential splice site (AGGC) in novel exons 3 and 7, which resulted in the generation of a GC-AG intron.

### Validation of *CEACAM5* mRNA expression levels

**Figure 3:**
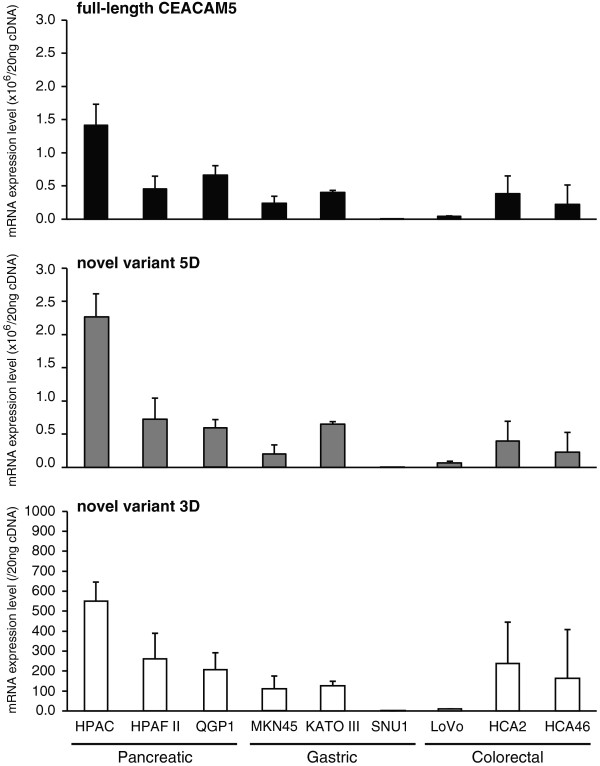
**Quantitative real-time (qRT)-PCR expression analysis of full-length and mutant *CEACAM5*.** The mRNA expression levels of full-length *CEACAM5* and novel variants 5D and 3D are shown in the upper panel, middle panel and lower panel, respectively. qRT-PCRs using specific primers was performed with 20 ng of cDNA templates synthesized from extracted mRNAs. Data are expressed as mean value ± S.D. in triplicate experiments.

Novel splice variants were detected in pancreatic, gastric, and colorectal cancer cells. To properly estimate the mRNA expression of these variants, we constructed a qRT-PCR assay for the quantification of *CEACAM5* transcripts. The specificity of qRT-PCR was first verified by gel electrophoresis and dissociation curve analysis (Additional file 2: Figure S1). As shown in Figure 1, all transcripts appeared to be highly expressed in the pancreatic cancer cell lines. The specificity of qRT-PCR was validated using these cells. Additionally, to confirm expression of novel transcripts in tumor tissues, we performed validation of qRT-PCR using commercialized total RNA derived from some gastrointestinal tissues. Amplicons were observed in a single band in electrophoresis, and the size of the bands was identical to the expected size. The melting curve analysis showed a single peak in all qRT-PCR assays. These results indicate that nonspecific PCR products were not amplified in the qRT-PCR. Standard curves were next generated from 10-fold serial dilutions of template DNA that were amplified from plasmids containing the sequences of the target transcripts (Additional file 3: Figure S2). The amplification efficiency of all transcripts was 97–111% (*R*^2^ > 0.990). These results indicate that the target-specific primers and the constructed template DNA provided accurate amplification in our qRT-PCR assay. We finally quantified the *CEACAM5* transcripts by using this validated assay (Figure 3). The mRNA expression profile of variants 5D and 3D in cancer cells was determined by this quantification analysis and electrophoresis of the RT-PCR amplicons. Additionally, these variants were expressed in colorectal normal tissue and some gastrointestinal tumor tissues (Additional file 4: Figure S3). The identified splice variants of *CEACAM5* were co-expressed with the full-length transcript without exception.

### Detection of CEA protein isoforms derived from novel splice variants

**Figure 4:**
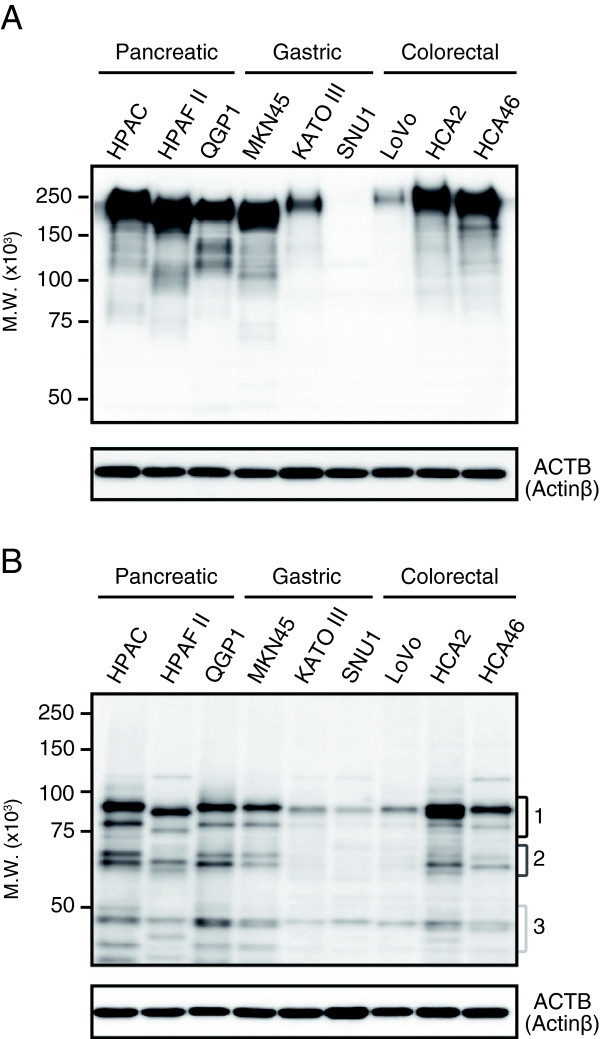
**Immunoblot detection of CEA in pancreatic, gastric, and colorectal cancer cells.** Cell lysate samples of each cell line (10 μg protein/lane) were analyzed by immunoblotting with the anti-CEA mouse monoclonal antibody (1:3,000 dilution), either directly **(A)** or after PNGase F treatment **(B)**. PNGase F treatment was undergone to cleave *N*-glycan chain from proteins. ACTB (actin-ß) was also detected as an internal loading control. The detected bands were separated into three regions, indicated as Regions 1, 2, and 3.

We also determined CEA protein expression in cancer cell lines. CEA is known to be highly *N*-glycosylated and to have different glycosylation patterns among cell lines [18,19]. As shown in Figure 4A, the main bands were detected around 200 kDa except for in SNU1, and a few bands were detected under 150 kDa. Full-length CEA and the isoforms are therefore considered to have been detected around 200 kDa and under 150 kDa, however, the practical molecular weight of these isoforms was unclear because of its large amount of *N*-linked oligosaccharides.

To detect CEA bands on the theoretical molecular weight calculated from the amino acid sequences, cell lysate samples were treated with PNGase F, which cleaves *N*-glycan chains from proteins. After PNGase F treatment, bands were clearly detected in three regions (Figure 4B). The theoretical molecular weight was as follows: CEA full-length, 80 kDa; isoform derived from variant 5D, 60 kDa; and isoform derived from variant 3D, 40 kDa. Considering these findings, the band detected in region 1 was considered to represent full-length CEA and the bands in regions 2 and 3 were considered to be the CEA isoforms derived from the novel splice variant 5D or 3D.

### Identification of CEA protein isoforms by LC-MS/MS analysis

To confirm that the lower bands (regions 2 and 3 in Figure 4B) detected by the CEA-specific antibody were novel CEA isoforms, proteins from each region treated with PNGase F were subjected to proteomic analysis. Table 1 shows the peptide sequences identified by the LC-MS/MS analysis. These data indicate that eleven tryptic peptides were identified from region 1 and the only protein that includes these sequences is full-length CEA. Moreover, a database search for the parameter, conversion of asparagine to aspartic acid confirmed that seven asparagine sites on these peptides were *N*-glycosylation sites.

**Table 1:** Peptide sequences identified by LC-MS/MS analysis

**Band region **^**†**^	**Sequence**	**Motif **^**‡**^
Region 1	KLTIESTPFNVAEGK	N
	QIIGYVIGTQQATPGPAYSGR	N
	SDLVNEEATGQFR	N
	TLTLFN^*^VTR	A1/A3^§^
	CETQNPVSAR	A1
	SDSVILNVLYGPDAPTISPLN^*^TSYR	A1-B1
	LQLSNDN^*^R	A2
	NSGLYTCQAN^*^NSASGHSR	B2
	LQLSNGN^*^R	A3
	AYVCGIQNSVSAN^*^R	A3
	ITPNNN^*^GTYACFVSNLATGR	B3
Region 2	KLTIESTPFNVAEGK	N
	SDLVNEEATGQFR	N
	TLTLFN^*^VTR	A1/A3^§^
	TLTLLSVTR	A2
Region 3	TLTLFN^*^VTR	A1/A3^§^
	ITPNNN^*^GTYACFVSNLATGR	B3

_*, conversion of asparagine to aspartic acid as a result of PNGase F treatment.

†, region detected by immunoblotting described in Figure 4.

**Figure 5:**
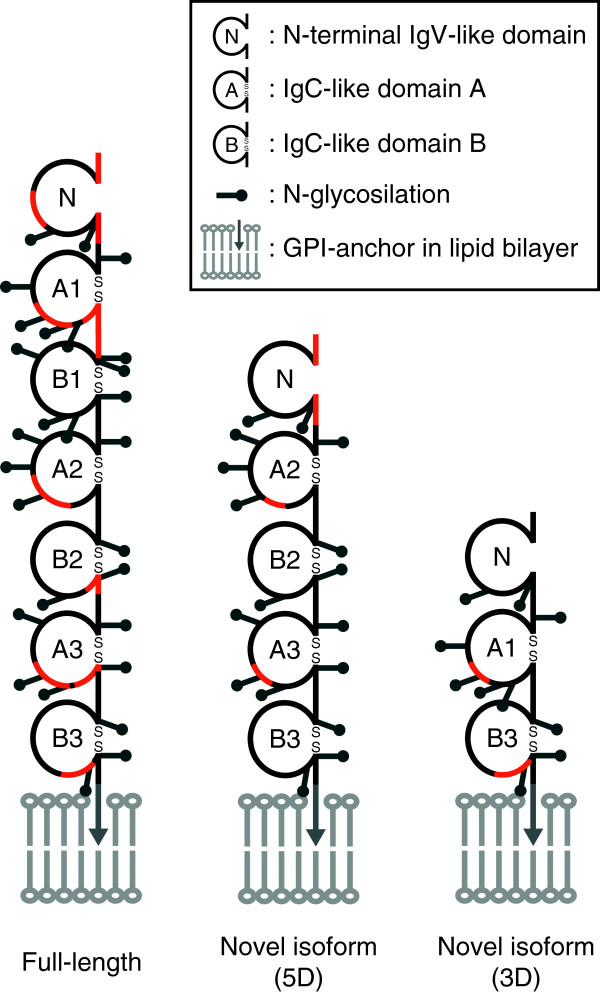
**Schematic illustrations of full-length CEA and novel isoforms.** Each motif (according to http://www.carcinoembryonic-antigen.de/index.html) is indicated as follows: N, amino-terminal IgV-like domains; A/B, IgC-like domains; lollipop, *N*-glycosylation sites predicted with consensus sequences (N-X-S/T, X ≠ P); arrowhead, glycosylphosphatidylinositol (GPI) linkage to the cell membrane. The location of peptides identified by LC-MS/MS analysis are indicated in red.

‡, the name of IgC/V-like domains described in Figure 5.

§, the same tryptic peptides on A1 and B3 domains.

Amino acid sequences of CEA isoforms, 5D and 3D, were predicted on the basis of DNA sequences (Additional file 5: Figure S4). Based on the resulting amino acid sequences and the structure of CEA [9,20-22], the domain motifs of the isoforms are illustrated in Figure 5. The full-length CEA consists of seven Ig-like domains containing an IgV-like domain (N) and six IgC-like domains (A or B), whereas isoform 5D and 3D consist of five or three Ig-like domains respectively; some IgC-like domains are deleted in these isoforms. Some peptide sequences of CEA were also identified from regions 2 and 3. These peptides identified by MS/MS analysis were located on domains with isoforms 5D and 3D but not on the deleted domains (Table 1 and Figure 5). These results indicate that the lower bands detected by the anti-CEA antibody around 60 kDa and 40 kDa represent CEA isoforms derived from the novel splice variants.

### Secretion of CEA protein to serum-free conditioned medium

**Figure 6:**
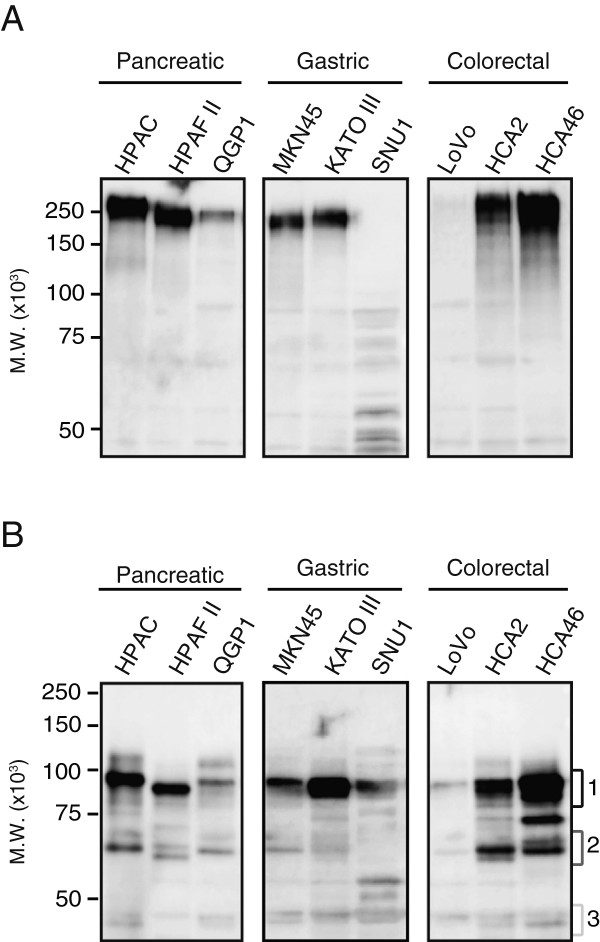
**Immunoblot detection of CEA in serum-free conditioned medium of pancreatic, gastric, and colorectal cancer cells.** Serum-free conditioned medium of each cell line was analyzed by immunoblotting with the anti-CEA mouse monoclonal antibody (1:1,000 dilution), either directly **(A)** or after PNGase F treatment **(B)**. The detected bands were separated into three regions, indicated as Regions 1, 2, and 3.

Since CEA is known to be secreted into the blood, we determined the form of CEA that was secreted into the conditioned medium. As shown in Figure 6A, the main band was detected around 200 kDa. After PNGase F treatment, the main band was detected around 80–90 kDa, which was similar to the size in the cell lysate samples (Figure 6B). In the LC-MS/MS analysis of serum-free conditioned medium after PNGase F treatment, the main band detected at region 1 was represented CEA (full-length), the band at region 2 was isoform 5D, and the band at region 3 was isoform 3D (data not shown). From other regions (e.g., HCA46 between regions 1 and 2), the CEA-related peptides were not detected. These results suggested that not only the full-length protein but also the isoforms of CEA were secreted to the medium.

## Discussion

Alternative splicing allows a single gene to generate multiple mRNAs that can be translated into diverse proteins [23]. Many transcripts have been predicted by *in silico* approaches and registered in public databases (e.g., Ensembl, http://www.ensembl.org) as candidate splice variants [24,25]. Recently, splice variants and their protein isoforms not registered in these databases were identified by transcriptome analysis [26-28]. Dorard et al. revealed that a novel HSP110 variant in colorectal cancer inhibits the function of wild-type proteins, which results in facilitation of apoptosis and increased chemosensitivity [28]. Thus, it is of importance to discover unpredictable splice variants and protein isoforms, and here, we focused on CEA and its isoforms that were not registered in databases.

The exon structure of many splice variants found in transcriptome analysis was determined using mRNA sequencing and potential GT-AG splice sites. Although we identified two novel splice variants of CEACAM5 (variant 5D and 3D), the splice site between exon 3 and 7 in variant 3D was not defined using this procedure (Figure 2A). To clarify the exon structure of variant 3D, we screened other potential splice sites in the unique sequence shared between exon 3 and 7, which resulted in identification of the specific splice site (AGGC). Because this identified site was present in the shared exons, the GC-AG intron is considered to be generated from pre-mRNA (Figure 2B). These findings raise the possibility that a part of the pre-mRNA derived from *CEACAM5* is processed by a U2-type spliceosome. Recently, Peng et al. reported another *CEACAM5* variant that does not include a major splice site between exons 2 and 5 [29]. This variant was not found in cancerous cells in our screening. The novel *CEACAM5* variants in this study were probably cancer-specific transcripts that were processed by unpredictable alternative splicing.

The *CEACAM5* splice variant involving exons 9 and 10 described in a previous report [30] were previously regarded as a PCR artifact [29]. To prevent the generation of artificial amplicons, we performed validation and quantification of *CEACAM5* transcript expression. Using the validated qRT-PCR assay, we found that the mRNA expression level of variant 5D was similar to that of the full-length transcript. However, in electrophoresis performed after RT-PCR, significant differences in expression levels were observed. We sequenced PCR products that were amplified using the primer pair used in Figure 1 and found several artifacts, i.e., electrophoresis bands at approximately 1,800 bp (data not shown). Therefore, we concluded that the difference between RT-PCR and qRT-PCR results likely reflects the presence of artificial amplicons.

Compared to the amino acid sequences of full-length CEA, no unique sequences were found in the novel CEA protein isoforms. Since the trypsinized peptide sequences of these isoforms are “shared” with those of the full-length protein, it is difficult to discuss the existence of isoforms based only on a typical MS/MS-based proteomic analysis. Some research groups have suggested that the combination of MS/MS-based proteomic analysis and protein separation based on molecular weight enables detection of protein isoforms that contain no unique sequences [31,32]. However, these approaches are not suitable for the detection of protein isoforms with inconsistency between molecular weight observed in protein separation and that calculated from the amino acid sequences. Most of secreted/membrane proteins undergo posttranslational modifications, and glycosylation especially affects the results of size-dependent protein separation. In the separation approach, such proteins are not detectable at the theoretical molecular weight calculated from the amino acid sequences. Indeed, CEA is a highly *N*-glycosylated protein. The practical molecular weight of full-length CEA is 180–200 kDa, even though the theoretical molecular weight is 76.8 kDa [33-35]. The novel isoforms also have a large amount of *N*-linked oligosaccharides, which result in difference between the practical and theoretical molecular weight. The inconsistency in the molecular weight could be resolved by deglycosylation. In the present study, we proposed at strategy that avoids the influence of *N*-glycosylation on protein separation through enzymatic deglycosylation.

Interestingly, mRNAs of CEA were not expressed in SNU1 cells, though the bands were slightly detected by anti-CEA antibody on SNU1 cells after PNGase F treatment (Figures 4 and 6). To gain more insight into CEA-expression on SNU1 cells, condition-optimized immunoblot detection (details in figure legends in Additional file 6: Figure S5) and protein identification by LC-MS/MS analysis were performed. Bands were detected on SNU1 samples (cell lysates and conditioned medium) regardless of the PNGase F treatment, however, CEA-related peptides were not identified from those band-detected regions (data not shown). These findings indicated that the bands detected on SNU1 by the anti-CEA antibody were non-specific bands. Accordingly, mRNA and protein of CEA were considered not to be expressed on SNU1 cells.

CEA in the blood was first observed in 1969 by Thomson et al. [3] and now is one of the most widely used tumor markers, however, the secreted forms and secretion mechanisms of CEA remains unclear. We found that CEA protein isoforms were secreted to the conditioned medium and that these isoforms always co-existed with the full-length CEA. Interestingly, the observed secretion pattern of CEA did not correlate with the expression pattern, as determined using a commercial anti-CEA antibody. Secretion of CEA isoform 5D is higher from colorectal cancer cell lines than from pancreatic and gastric cancer cell lines. These findings suggest that CEA protein isoforms may be secreted by the specific mechanisms from certain cancer cells.

The novel CEA isoforms contain a Ig-V like domain and some IgC-like domains; two or four IgC-like domains are deleted in those isoforms (Figure 5). IgC-like domains are required for functionality of CEACAM family proteins as homophilic and heterophilic intercellular adhesion molecules [36]. CEA-CEA homophilic interaction between an IgV-like domain and six IgC-like domains occurs in an antiparallel reciprocal manner, which is unique in this family, and it can directly influence cancer invasion and metastasis [37-39]. Those short CEA isoforms might inhibit full-length CEA-CEA homophilic interactions or mediate the heterophilic interaction with other CEACAM family proteins, leading to the influence of CEA-related intracellular signaling events. Limitations of this study are there because of poor functional analysis of novel CEA isoforms; further studies such as *in vivo* experiments and direct detection of those isoforms, are necessary to investigate the physiological significance of CEA isoforms.

## Conclusions

The present study identified novel splice variants/protein isoforms of CEA, and provided direct evidence that the protein isoforms were not only co-expressed with full-length CEA but also co-secreted into culture medium by gastrointestinal cancer cell lines. CEA is a well-known serum tumor marker, however, because of its low sensitivity and specificity, the main use of serum CEA determinations is currently in the postsurgical surveillance. Discrimination between full-length CEA and its isoforms may improve the clinical utility of CEA as a tumor marker.

## Availability of supporting data

The protein coding sequences of *CEACAM5* splice variants 5D and 3D are deposited in DNA Data Bank of Japan (DDBJ, http://www.ddbj.nig.ac.jp/index-e.html), which are accessible through the DDBJ accession number AB852566 and AB852567, respectively.

## Competing interests

The authors declare that they have no competing interests.

## Authors’ contributions

KH and KWN designed the study, interpreted the data, and drafted the manuscript. KO, NS, and TM were scientific leads and assisted in designing the study. KY and TM provided reagents, materials, and analysis tools. All authors read and approved the final manuscript.

## Supplementary Material

Additional file 1: Table S1 List of sequences of RT-PCR, quantitative RT-PCR, and DNA sequencing primers. Click here for file

Additional file 2: Figure S1 Specificity verification for quantitative real-time (qRT)-PCR assay. Click here for file

Additional file 3: Figure S2 Standard curves generated from 10-fold serial dilutions of recombinant plasmid DNA. Click here for file

Additional file 4: Figure S3 Quantitative qRT-PCR expression analysis of full-length and mutant CEACAM5 in normal and tumor tissues. Click here for file

Additional file 5: Figure S4 Exon structure of the two novel splice variants and amino acid sequences of variants-derived protein isoforms. Click here for file

Additional file 6: Figure S5 Immunoblot detection of CEA in SNU1 cells. Click here for file
