# Supplementary material for: Novel protein isoforms of carcinoembryonic antigen are secreted from pancreatic, gastric and colorectal cancer cells
Source: BMC Res Notes. 2013 Sep 26;6:381. doi: 10.1186/1756-0500-6-381 (PMC3850884; doi:10.1186/1756-0500-6-381)
Supplement: Additional file 1: Table S1 — List of sequences of RT-PCR, quantitative RT-PCR, and DNA sequencing primers. [file 1756-0500-6-381-S1.doc]

**Table S1** DNA sequences of RT-PCR, quantitative RT-PCR, and DNA sequencing primers

| Target | Sense primer (location) | Antisense primer (location) |
| --- | --- | --- |
| RT-PCR and DNA sequencing | | |
| all *CEACAM5* | ccgcatacagtggtcgagagata (exon 2) | tatatcagagcaaccccaaccag (exon 9) |
|  | tccccctcatacacctattaccg (exon 6) | ggcaggtatagagtccgctgttc (exon 6) |
|  |  | tccttggtaaagaagcaattttagac (exon 10) |
| *ACTB* | tggcacccagcacaatgaa (exon 5) | ctaagtcatagtccgcctagaagca (exon 6) |
| Quantitative RT-PCR | | |
| NM_004363 | gtgccaagcccataactcaga (exon 4) | ttggagttgttgctggtgatg (exon 5) |
| novel variant 5D | atgcatccctgctgatcca (exon 2) | ttggagttgttgctggtgatg (exon 5) |
| novel variant 3D | ctacctgtggtgggtaaacaatca (exon 3) | ctgcggtttgcactcactga (exon 7) |
