# Supplementary material for: Novel protein isoforms of carcinoembryonic antigen are secreted from pancreatic, gastric and colorectal cancer cells
Source: BMC Res Notes. 2013 Sep 26;6:381. doi: 10.1186/1756-0500-6-381 (PMC3850884; doi:10.1186/1756-0500-6-381)
Supplement: Additional file 2: Figure S1 — Specificity verification for quantitative real-time (qRT)-PCR assay. [file 1756-0500-6-381-S2.pdf]

Figure S1

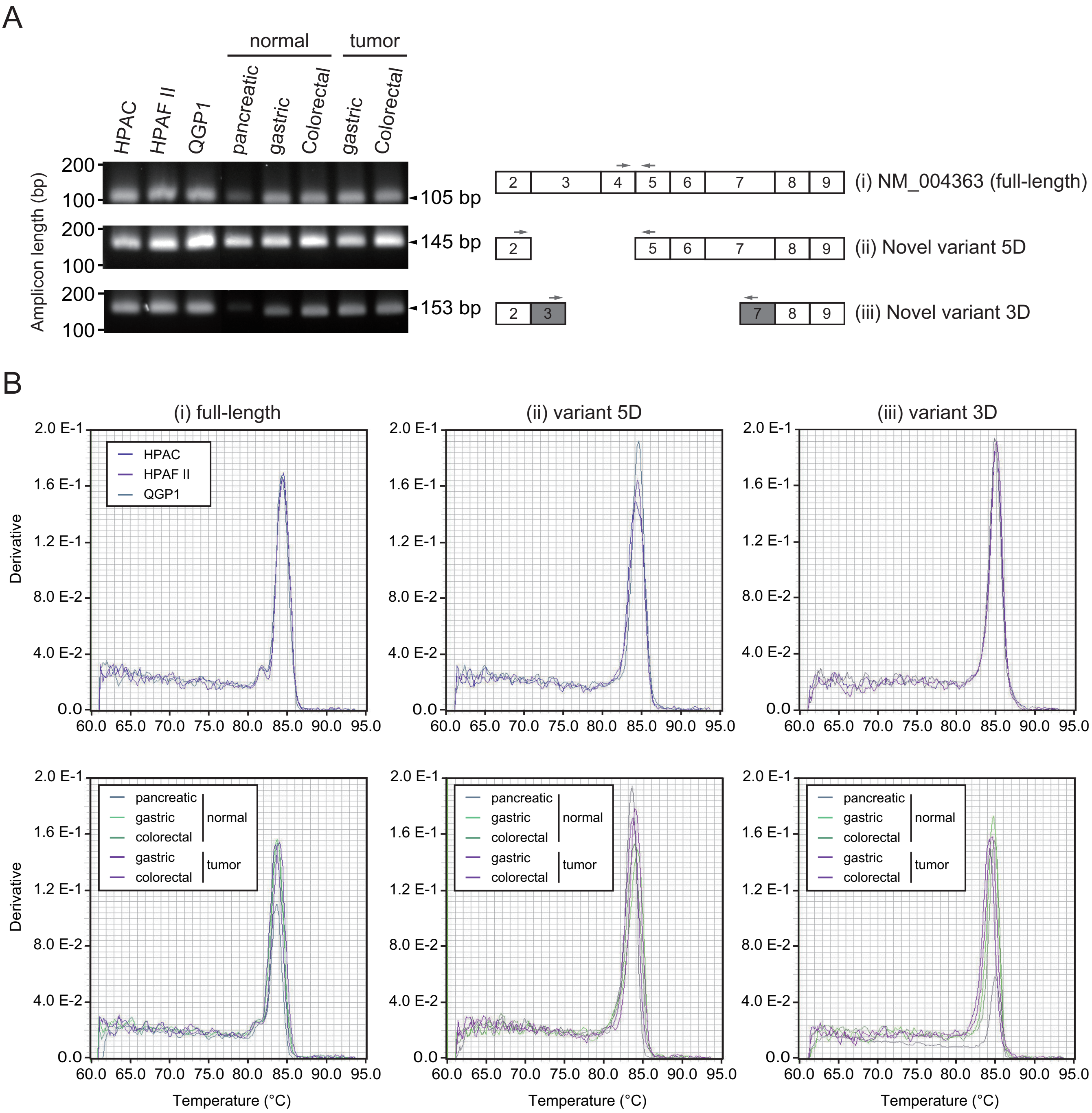

Figure S1. Specificity verification of quantitative real-time (qRT)-PCR assay. (A) Agarose gel electrophoresis analysis of qRT-PCR amplicons. Electrophoresis images of amplicons in pancreatic cancer cells and gastrointestinal tisseus are shown in left panel. The amplicon sizes of full-length CEACAM5 (i), variant 5D (ii) and variant 3D (iii) were 105, 145 and 153 bp, respectively. Schematic diagram of exon structures corresponding to the detected amplicons are represented in the right panel. Primer positions for qRT-PCR are indicated by gray arrows. (B) Melting curve analysis of qRT-PCR amplicons (i~iii). First-derivative curves of fluorescence decay derived from dissociation of intercalator and DNA were plotted versus temperature. The curves of cell lines and tissues are shown in upper and lower panels, respectively.
