# Supplementary figures and images for: Novel protein isoforms of carcinoembryonic antigen are secreted from pancreatic, gastric and colorectal cancer cells
Source: BMC Res Notes. 2013 Sep 26;6:381. doi: 10.1186/1756-0500-6-381 (PMC3850884; doi:10.1186/1756-0500-6-381)

**full-length CEACAM5**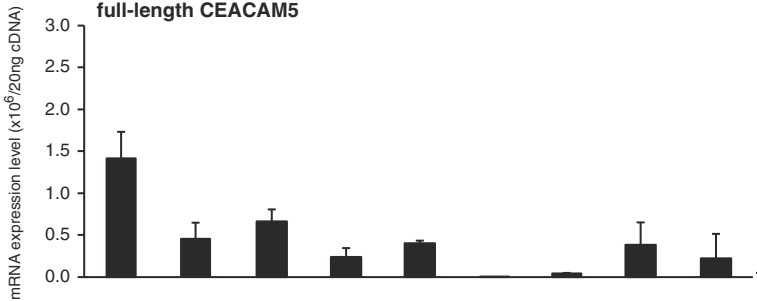**novel variant 5D**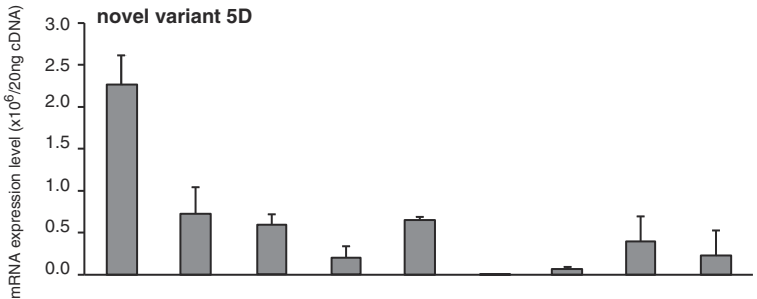**novel variant 3D**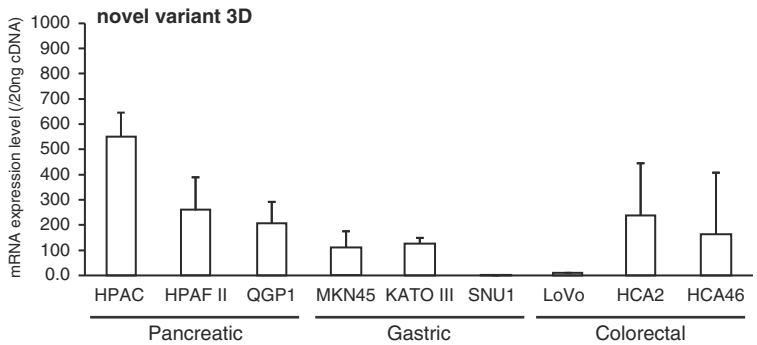

Supplement: Additional file 3: Figure S2 — Standard curves generated from 10-fold serial dilutions of recombinant plasmid DNA. [file 1756-0500-6-381-S3.pdf]

**A**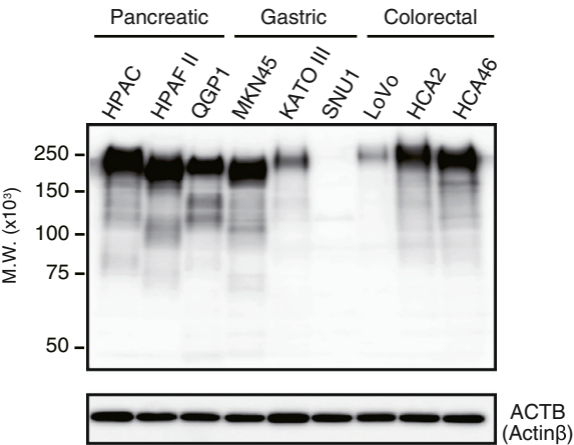**B**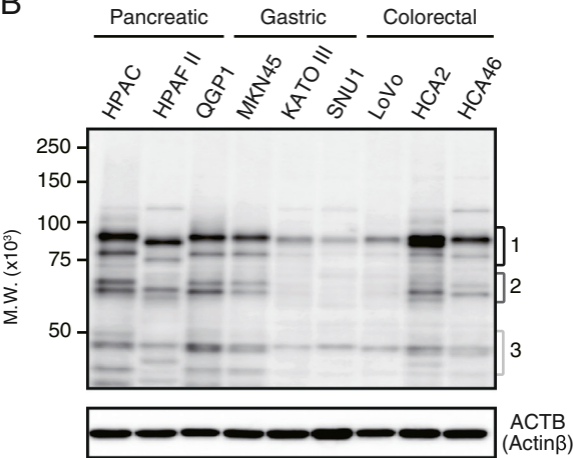

Supplement: Additional file 4: Figure S3 — Quantitative qRT-PCR expression analysis of full-length and mutant CEACAM5 in normal and tumor tissues. [file 1756-0500-6-381-S4.pdf]

**A**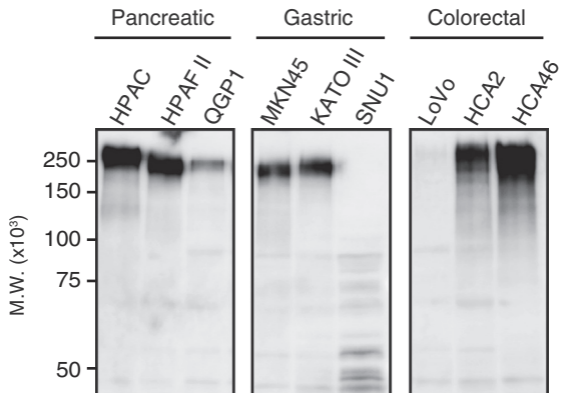**B**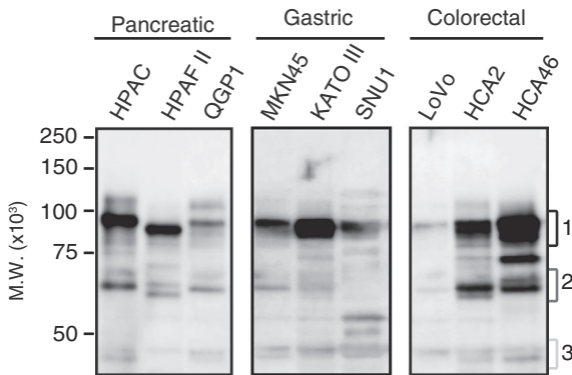

Supplement: Additional file 6: Figure S5 — Immunoblot detection of CEA in SNU1 cells. [file 1756-0500-6-381-S6.pdf]
