# Supplementary material for: Novel protein isoforms of carcinoembryonic antigen are secreted from pancreatic, gastric and colorectal cancer cells
Source: BMC Res Notes. 2013 Sep 26;6:381. doi: 10.1186/1756-0500-6-381 (PMC3850884; doi:10.1186/1756-0500-6-381)
Supplement: Additional file 5: Figure S4 — Exon structure of the two novel splice variants and amino acid sequences of variants-derived protein isoforms. [file 1756-0500-6-381-S5.pdf]

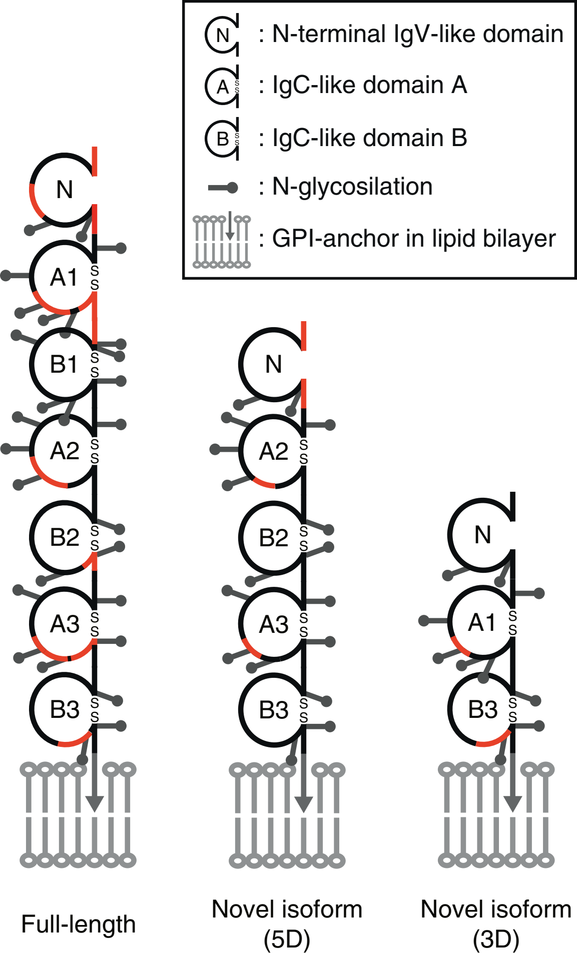

N
: N-terminal IgV-like domain
A
: IgC-like domain A
B
: IgC-like domain B
: N-glycosilation
: GPI-anchor in lipid bilayer
N
A1
B1
A2
B2
A3
B3
N
A2
B2
A3
B3
N
A1
B3
S S
Full-length
Novel isoform (5D)
Novel isoform (3D)
